# Supplementary material for: The effect of Iran’s health transformation plan on hospital performance: Kerman province
Source: PLoS One. 2021 Feb 17;16(2):e0247155. doi: 10.1371/journal.pone.0247155 (PMC7888626; doi:10.1371/journal.pone.0247155)
Supplement: Attachment — Submitted filename: Response to Reviewers.docx [file pone.0247155.s001.docx]

Dear editor in chief

Thank you for your invaluable comments and guidance in improving this manuscript. It should be noted that based on the Reviewer’s comments and according to Journal requirements, we made some changes in manuscripts that are specified in text. We presented the responses to reviewers’ comments separately in the following table. I as the corresponding author on behalf of all authors express our readiness to do any further revision seems necessary by the journal.

| Response | Comments | Reviewer |
| --- | --- | --- |
| Thanks for your comment. In our revised version, we provide more detail about hospital data. A total of 24 hospitals were entered to study, 5 of which were excluded from the final analysis due to lack of sufficient data (Please see page 5, lines 86-89). | 1. In the methods section, the authors mentioned that 19 selected hospitals are included in the study. What is the total number of the hospitals in the province? Is the 19 hospitals representative number or not? | Reviewer#1 |
| Thanks for your comment. Although some variables changed significantly after HTP, the physician variable was not significant and thus the efficiency and productivity changes after HTP were not significant. We discuss this in the Discussion section of the revised manuscript (Please see pages 12 and 13, lines 216 and 240-242).  We also performed a regression to find that the physician variable could be more important than the other variables( Please see the figure below).   | 2. There is no explanation why 1) the average efficiency was not significant. Can you explain this result in terms of the results of the table 1 which includes significant tests with p-value <0.05. |  |
| Thanks for your comment. We have changed the Table 2 as suggested by reviewer #1.  Statistical tests were performed for productivity changes before and after HTP and were reported in Table 3. | 3. Table 2 is not very clear. It does not include any significant results, that is technical efficiency has no significant change. Can you connect table 2 results with table 3? Is there statistical significant change of the Malmquist productivity index between years? |  |
| Thanks for your comment. We first assessed the normality and abnormality of the statistical distribution of data in each scenario using the Kolmogorov – Smirnov test; then, we measured the efficiency and productivity changes by appropriate statistical tests ( Paired t-test or Wilcoxon). In this study, the efficiency data of university hospitals had a normal distribution, so we used the paired t-test to measure changes in efficiency before and after HTP.  However, the efficiency data of non-university hospitals were abnormally distributed, and the Wilcoxon test was used. The efficiency data of all hospitals were also abnormally distributed. We have explained more in the revised version (Please see page 7, lines 130-136). | 4. In table 2, university hospitals efficiency is done with Paired t -test. The non university hospitals test is wilcoxon. When you test total hospitals you use Wilcoxon. Don't you think this is inconsistency? in the first case you have normality assumption and the other 2 categories you use non parametric test. Please explain!!! |  |
| Thanks for your comment. As suggested, we have added details on the importance of decision-making in the health system (Please see pages 3 and 4, lines 59-67). | 1. The introduction section needs to be clarified for the readers for better understanding. There is need of more references so far decision making system is concerned. Literature are available in the context of global level in decision making. | Reviewer#2 |
| Thanks for your comment. We have added more details in the revised version (Please see pages 5-7, lines 101-109 and 132-136). | 2. In methodology the authors should explain the procedures in detail so that the study can be replicated elsewhere. for example DEA method needs more specification. Data analyse procedures also need details explanation. |  |
| Thanks for your comment. We have changed Tables 2 and 3 as suggested by reviewer #2. | 3. In result table 1 is understandable But, table 2 and 3 need explanation and scientific interpretation of the results. there is also need of clarity do the understanding of the readers. |  |
| Thanks for your comment. As suggested, the discussion section is updated in the revised version of the paper. | 4. the discussion section needs complete overwhelming. Similar results are to be interpreted with study of more literature. Important results from the tables are still missing in discussion section |  |
