# Supplementary material for: The effect of Iran’s health transformation plan on hospital performance: Kerman province
Source: PLoS One. 2021 Feb 17;16(2):e0247155. doi: 10.1371/journal.pone.0247155 (PMC7888626; doi:10.1371/journal.pone.0247155)
Supplement: Attachment — Submitted filename: Response to Reviewers.docx [file pone.0247155.s002.docx]

Dear editor in chief

Thank you for your invaluable comments and guidance in improving this manuscript. It should be noted that based on the Reviewer’s comments and according to Journal requirements, we made some changes in manuscripts that are specified in text. We presented the responses to reviewers’ comments separately in the following table. I as the corresponding author on behalf of all authors express our readiness to do any further revision seems necessary by the journal.

| Response | Comments | Reviewer |
| --- | --- | --- |
| Thanks for your comment. We added two figures in our revised version so that the reader can understand the changes in productivity and efficiency better (please see figures 1 and 2 and see page 11, lines 193-196). | I think if you use graphical representation for the productivity (eg histograms or boxplots) before and after HTP, the readers would get better understanding of your variables. | Reviewer#1 |
| Thanks for your comment. We added standard errors of productivity numbers for different scenarios (please see table 3). | Can you report the standard error of the productivity numbers in table 3? This could tell us important information about the variability of productivity for the suggested 4 categories |  |
| Thanks for your comment. We added some studies to compare with the present study in the discussion section (please see page 12, lines 208-210 and page 13, lines 234-236). | 1. The article need more discussion to make the study comparable with others. | Reviewer#2 |
| Thanks for your comment. We searched Scopus and Pubmed databases for the most recent studies related to this research, and two studies were added to the introduction section (please see page 4, lines 80-82). | 2. the reference articles have to be from very high authoritative sources. New articles should be added in introduction section to make the technicalities understandable. |  |
| Thanks for your comment. We provided more details about statistical tests in our revised version (please see page 9, lines 181, 196, and page 10, line 197). | 3. The tables especially paired test tables have to be explained for understanding. |  |
